# A framework for real-time image detection of bioaerosols

**DOI:** 10.1038/s41598-025-32744-x

**Published:** 2025-12-29

**Authors:** Ryne A. Juidici, Yan Ye, Francisco J. Romay, Miles Owen, Qisheng Ou, David Y. H. Pui

**Affiliations:** 1 Mechanical Engineering, University of Minnesota, 111 Church St SE, Mechanical Engineering Building, Minneapolis, MN 55455 USA; 2 Y2Y Technology, Santa Clara, CA 95052 USA; 3 U.S. Army Primary Standards Laboratory, Redstone Arsenal, AL USA

**Keywords:** Biological techniques, Biophysics, Optics and photonics, Physics

## Abstract

Starting from the elastic light scattering and the induced fluorescence emission from airborne particles and extending to the counting and differentiation of the associated signals, a framework is proposed to describe the detection of bioaerosols using an image sensor. For validation, monodisperse NaCl particles were generated to mimic abiotic particles, while monodisperse 1% mass riboflavin particles were generated to mimic biotic particles. By challenging a prototype sensor with these particles, the exposure time, particle speed, and signal-to-noise ratio were shown to be critical parameters for detection. Additionally, it was displayed that the induced fluorescence emission can be isolated from the elastic light scattering by using a well-selected long-pass filter. Furthermore, correlating the color of the captured signals to an induced fluorescence contribution was shown to be a potential avenue of differentiation between biotic and abiotic particles. It is predicted that this color differentiation method can distinguish between a near-continuous range of visible induced fluorescence emission wavelengths, giving the ability to distinguish individual fluorophores from one another using simple filtering and a single detector. This framework will be used to further optimize the image-based bioaerosol sensor evaluated here.

**Supplementary Information:**

The online version contains supplementary material available at 10.1038/s41598-025-32744-x.

## Introduction

Real-time knowledge of bioaerosol concentrations and identities is an important piece of information in many monitoring applications. For an example, the presence of aerosolized mold has been previously associated to varying degrees with allergic and asthmatic symptoms^1,2^. Real-time detection of these aerosolized mold particles can allow for the timely implementation of improved ventilation or other remediation efforts. For a more time-sensitive example, real-time detection of the bacteria *Bacillus anthracis* could help with the immediate evacuation of a targeted area^3^. Many different methods and instruments have been developed to achieve real-time detection of bioaerosols and address these applications^4,5^.

There are multiple commercial instruments that have varying capabilities for real-time monitoring of bioaerosols including, but not limited to the ENVI BioScout (Bertin Environics, Finland), the Wideband Integrated Bioaerosol Sensor (WIBS, Droplet Envea Group, United States), and the BioTrak Real-Time Viable Particle Counter (TSI, United States)^6,7^. These instruments mainly rely upon induced fluorescence to differentiate bioparticles from abiotic particles. Induced fluorescence of bioparticles occurs due to the presence of fluorophores. These fluorophores can absorb and then release a photon, assuming sufficient excitation. The released photons will have a longer wavelength than the original photons^8^. Most bioparticles contain some combination of these fluorophores. Induced fluorescence can also be present for certain abiotic particles, leading to the potential for false positives when relying solely upon this property for detection^9–11^. Despite this, induced fluorescence is widely used as a trigger detector for the presence of bioaerosols. Once triggered, more time-intensive methods of analysis can be completed for detailed verification and identification.

Due to their high sensitivity, many bioaerosol instruments focused on induced fluorescence detection use photomultiplier tubes as their main optical component^4,6,7^. For instruments relying upon photomultiplier tubes or single photodetectors to quantify the induced fluorescence signal, they are limited to interpreting the presence of particles as electrical pulses. This limits the information that can be gained without using complex optical systems. An image sensor is more effective at resolving simultaneous particle scattering/emission events due to capturing the spatial distribution of signals. Multiple particles can pass through the beam at a single time and still be differentiated as separate events. A single photodetector or photomultiplier tube would need to differentiate these events using solely the electrical signal or ensure minimal coincidence events. Additionally, the proposed framework will detail the use of the signal color to identify an induced fluorescence contribution while preserving a portion of the elastic scattering. The preservation of the elastic signal could allow for the detection and differentiation of both abiotic particles and bioparticles with a single sensor and simple filtering. To identify a similar amount of information, a single photodetector or photomultiplier tube system would either require a detector with multiple channels or several separate detectors, each configured for a different wavelength range. In theory, using an image sensor for the detection of bioaerosols allows for simpler optical setups as more information is readily available and recorded. This benefit comes at the cost of reduced sensitivity and higher computational requirements for detection.

Image sensors have has been applied previously for the detection of bioparticles^12–17^. All of the referenced instruments use some form of image sensor to detect bioparticles; however, they may also utilize unique techniques, in addition to or other than induced fluorescence, to differentiate bioparticles from abiotic particles, such as holography, fluorescent dyes, and spectral grating^12–17^. Additionally, many of the referenced instruments require the deposition of particles onto a substrate/surface before detection, whereas the goal for this prototype sensor is to keep the particles airborne^12–16^.

The proposed framework will focus solely upon using induced fluorescence emission as a marker for bioparticles, as the goal is to minimize the complexity and cost of the associated prototype sensor. The detection setup described here is an expansion of the previously proposed method by Ye et al.^18^. It is believed that the simple optical setup of the proposed framework and prototype sensor offer the reduced complexity and cost desired, compared to the previously mentioned approaches. Furthermore, by maintaining the airborne state of the challenged particles, the prototype sensor design does not require a consumable element, such as substrates or a working fluid. As far as the color differentiation approach that will be detailed, this method obtains similar information to the techniques used by Huffman et al. 2016 (by extension Swanson and Huffman), and Zhang et al. about the induced fluorescence contribution but forgoes the use of spectral grating which complicates the optical setup and spreads the induced fluorescence emission over a larger array of pixels^12,13,17^. This is at the cost of reduced information as the full spectrum of emitted light will not be known, only the simplified information presented by the signal color. For a task focused on bioaerosol identification (i.e. differentiating mold particles from pollen particles), the aforementioned information may be required; however, the focus here is general trigger detection of bioaerosols.

The scope of this report will be the introduction of a framework for the real-time image detection of bioaerosols. The objective of this framework is to mathematically describe the detection of airborne bioparticles using an image sensor and induced fluorescence, starting from the interaction of particles with an incident light source and extending to the counting and differentiation of the associated signals in a recorded image. While some of the general principles of this framework have been implemented in the previously mentioned studies, it is believed that this framework is unique in its description of these principles and aims to provide a clear summary of the interactions involved. To validate the proposed framework, the experimental results obtained using a prototype sensor will be presented. While the prototype sensor is still under development and requires further optimization before the limits of the method can be verified, these experimental results serve to confirm and describe the general detection trends associated with the framework. Both the limitations currently observed with the method and the expected limitations with the method are discussed to provide a baseline for what can be accomplished. The proposed framework will be used to guide the optimization of the prototype sensor and serve as a starting point for the description of similar methods.

## A framework for detection and differentiation

### Elastic light scattering

The light scattered and emitted from a bioparticle passing through an incident light source will be a combination of the elastic light scattering and induced fluorescence emission, assuming sufficient excitation. For elastic light scattering, Rayleigh scattering is typically assumed for particles much smaller than the wavelength of the incident light^19^. This form of scattering, assuming a spherical particle and unpolarized incident light, is described by Eq. (1).

1 $$\:{I}_{p,e,\lambda}\left(\theta\right)=\frac{{I}_{0}*{\pi}^{4}*{{d}_{p}}^{6}}{8*{R}^{2}*{\lambda}_{0}^{4}}*{\left(\frac{{m}^{2}-1}{{m}^{2}+2}\right)}^{2}*\left(1+co{s}^{2}\left({\uptheta}\right)\right)$$

$\:R$ is the distance from the particle, $\:{d}_{p}$ is the particle diameter, $\:{{\lambda}}_{0}$ is the incident light wavelength, $\:m$ is the refractive index of the particle, $\:{\uptheta\:}$ is the angle between the scattering direction and the incident light direction, $\:{I}_{0}$ is the intensity of the incident light, and $\:{I}_{p,e,\lambda}$ is the intensity of the elastically scattered light. The $\:\lambda\:$ subscript denotes the wavelength associated with that intensity. For elastic scattering, $\:\lambda\:$ is equal to $\:{\lambda}_{0}$. If the wavelength of the incident light is not significantly longer than the diameter of the particle, Mie scattering must be considered, described by Eq. (2) and assuming once again a spherical particle and unpolarized incident light.

2 $$\:{I}_{p,e,\lambda}\left(\theta\right)=\frac{{I}_{0}*{\lambda}_{0}^{2}*({i}_{1}+{i}_{2})}{8*{\pi}^{2}*{R}^{2}}\:$$

$\:{i}_{1}$ is the perpendicular polarization parameter, and $\:{i}_{2}$ is the parallel polarization parameter^19^. These two parameters are functions of the refractive index of the particle, the size of the particle, the wavelength of the incident light, and the scattering angle. For brevity, only the elastic scattering of unpolarized light is explicitly covered here; however, the above equations can be substituted with expressions for the elastic scattering of polarized incident light.

**Fig. 1:**
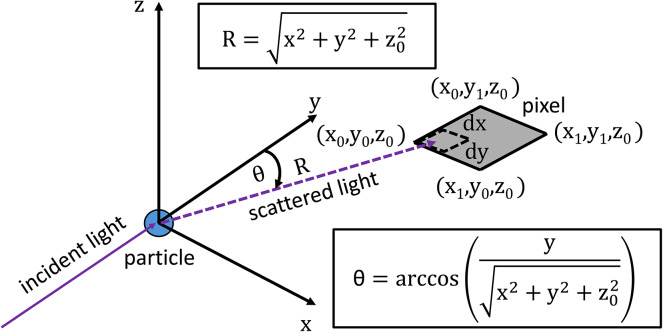
The assumed geometry of the pixel-particle system with the incident light pointed in the positive y-direction and a horizontal, rectangular pixel at a height of $\:{z}_{0}$. For the consideration of polarized light sources, this geometry will need modification as the elastic scattering will also be dependent on the angle between the polarization and the pixel.

In the proposed setup, the scattered light will fall upon an array of pixels, assumed to be rectangular. The radiant power delivered to a given pixel can be found by considering the geometry of the pixel-particle system. For simplicity and generality, the use of an imaging lens will not be considered directly in the geometry of the pixel-particle system. For the simplified geometry, the particle will be placed at the origin, the incident light will be a ray pointed in the positive y-direction, and the pixel will be placed horizontally at a fixed height of $\:{z}_{0}$ in the positive z-direction, as shown in Fig. 1.

With this geometry, $\:R$ is the radius from the origin, and $\:\theta\:$ is the same scattering angle as defined in Eqs. (1) and (2) . These two quantities can be described solely by the spatial position of the pixel relative to the particle as shown in Fig. 1. It will also be assumed that only the component of light normal to the pixel surface will contribute to the radiant power the pixel receives. Depending on the properties of the pixel, it may be a fair assumption to consider the full contribution of light reaching the pixel, rather than just the normal component; however, considering only the normal component serves as a more conservative assumption. Using this geometry and the given assumptions, the radiant power the pixel receives from the elastic light scattering is given by Eq. (3).

3 $$\:{P}_{p,e,\lambda,pixel}=\int\limits_{{x}_{0}}^{{x}_{1}} \int\limits_{{y}_{0}}^{{y}_{1}}{I}_{p,e,\lambda}\left(x,{y,z}_{0}\right)*\frac{{z}_{0}}{\sqrt{{x}^{2}+{y}^{2}+{z}_{0}^{2}}}dy\:dx$$

$\:{P}_{p,e,\lambda,pixel}$ is the radiant power a pixel receives from the elastic light scattering of a given particle.

The contribution from the elastic scattering of the medium must also be considered. This is described in detail in Ye & Pui^20^. Each of the molecules within the illuminated medium will elastically scatter the incident light according to Eq. (1), assuming that the molecular diameter of the medium is much smaller than the incident wavelength. The radiant power to the pixel from the medium can be found using Eq. (3) as well. The framework described in this paper will assume that the interference between the medium and the particle scattering, described in Ye & Pui, can be neglected^20^. By neglecting this interference, the light reaching a pixel from a given particle and the surrounding medium can be represented by a linear relationship that follows the superposition principle. In this manner, the effect of two or more overlapping waves at any point in space is the linear effect of the individual waves at that point.

### Induced fluorescence emission

The induced fluorescence emission involves a complex relationship that requires consideration of the excitation wavelength, the excitation intensity, the particle geometry, and the particle composition. Hill, et al. 2015 describes this relationship using a fluorescence cross section, $\:{C}_{f}$^21,22^. The total intensity of the induced fluorescence emission from a particle in all directions can be described by Eq. (4). For details on the calculation of the fluorescence cross-section, refer to Hill et al. 2013 and Hill et al. 2015.

4 $$\:{P}_{p,f,\lambda,full\:angle}={C}_{f}*{I}_{0}\:where\:{C}_{f}={C}_{f}({d}_{p},{\lambda}_{0},\dots\:)$$

The induced fluorescence emission given in Eq. (4) is only valid for full angle collection. The geometry in Fig. 1 does not fulfill this condition, so a modification must be made. The framework described by Hill et al. 2015 does not give an angular dependence, so one can only assume a generic dependence on the pixel position and the total fluorescence emission to describe the induced fluorescence emission reaching a given pixel. This can be expressed by Eq. (5).

5 $$\:{P}_{p,f,\lambda,pixel}=\int\limits_{{x}_{0}}^{{x}_{1}} \int\limits_{{y}_{0}}^{{y}_{1}}{{I}_{p,f,\lambda}(I}_{0},{C}_{f},x,y,{z}_{0})*\frac{{z}_{0}}{\sqrt{{x}^{2}+{y}^{2}+{z}_{0}^{2}}}dy\:dx$$

$\:{P}_{p,f,\lambda,pixel}$ is the radiant power a pixel receives from the induced fluorescence emission of a given particle.

### Total radiant power reaching a pixel

With the assumption of negligible interference, the total radiant power reaching a pixel can be considered as the superposition of the elastic light scattering from the particle, $\:{P}_{p,e,\lambda,pixel}$, the induced fluorescence emission from the particle, $\:{P}_{p,f,\lambda,pixel}$, and the background signal from sources of light other than the particles themselves, $\:{P}_{b,\lambda,pixel}$. The elastic light scattering from the medium is contained within this background radiant power term.

Until this point, the radiant power contributions have been described at a singular wavelength; however, the total radiant power can be considered as a discrete sum of radiant powers at key wavelengths, given in Eqs. (6)–(8).

6 $$\:{P}_{p,e,pixel}=\:\sum\limits_{i\:=1}^{n}{P}_{p,e,\lambda,pixel}\left({\lambda}_{i},{\lambda}_{i}+\varDelta\lambda\right)=\sum\limits_{i\:=1}^{n}\frac{{P}_{p,e,\lambda,pixel}\left({\lambda}_{i},{\lambda}_{i}+\varDelta\lambda\right)}{\varDelta\lambda}*\varDelta\lambda$$

7 $$\:{P}_{p,f,pixel}=\:\sum\limits_{i\:=1}^{n}{P}_{p,f,\lambda,pixel}\left({\lambda}_{i},{\lambda}_{i}+\varDelta\lambda\right)=\sum\limits_{i\:=1}^{n}\frac{{P}_{p,f,\lambda,pixel}\left({\lambda}_{i},{\lambda}_{i}+\varDelta\lambda\right)}{\varDelta\lambda}*\varDelta\lambda$$

8 $$\:{P}_{b,pixel}=\sum\limits_{i\:=1}^{n}{P}_{b,\lambda,pixel}\left({\lambda}_{i},{\lambda}_{i}+\varDelta\lambda\right)=\sum\limits_{i\:=1}^{n}\frac{{P}_{b,\lambda,pixel}\left({\lambda}_{i},{\lambda}_{i}+\varDelta\lambda\right)}{\varDelta\lambda}*\varDelta\lambda$$

n is the number of key wavelengths that must be considered, and $\:\varDelta\lambda$ represents a small interval of wavelengths. To represent this as a continuous integral over a range of wavelengths, a spectral power distribution can be defined by Eq. (9).

9 $$\:{S}_{i}\left(\lambda\right)=\lim_{\varDelta\lambda \rightarrow 0}\frac{{P}_{i}(\lambda,\lambda+\varDelta\lambda)}{\varDelta\lambda}\:$$

The $\:i$ subscript is a placeholder for the subscript of the three different contributions, and $\:{S}_{i}$ is the corresponding spectral power distribution for each contribution. The total radiant power can then be described as the integration of a spectral power distribution across the wavelength range of interest.

10 $$\:{P}_{p,e,pixel}\:=\:\int\limits_{{\lambda}_{i}}^{{\lambda}_{f}}{S}_{p,e,pixel}\left(\lambda\right)\:d\lambda\:$$

11 $$\:{P}_{p,f,pixel}\:=\:\int\limits_{{\lambda}_{i}}^{{\lambda}_{f}}{S}_{p,f,pixel}\left(\lambda\right)\:d\lambda\:$$

12 $$\:{P}_{b,pixel}\:=\:\int\limits_{{\lambda}_{i}}^{{\lambda}_{f}}{S}_{b,pixel}\left(\lambda\right)\:d\lambda\:$$

$\:{\lambda}_{i}$ is the shortest wavelength of interest, and $\:{\lambda}_{f}$ is the longest wavelength of interest. Additionally, there are various physical interactions that reduce the radiant power reaching the image sensor, such as the scattered light interacting with a filter applied in line with the image sensor. Many of these factors have some dependence on the wavelength of the light, especially those associated with optical filters. Equations (13)–(15) allow these physical interactions to be considered in the previously described integrations.

13 $$\:{P}_{p,e,pixel}\:\:=\:\int\limits_{{\lambda}_{i}}^{{\lambda}_{f}}\:\left(\prod\limits_{i=1}^{m}{f}_{i}\left(\lambda\right)\right)*{S}_{p,e,pixel}\left(\lambda\right)\:d\lambda\:$$

14 $$\:{P}_{p,f,pixel}=\:\int\limits_{{\lambda}_{i}}^{{\lambda}_{f}}\left(\prod\limits_{i=1}^{m}{f}_{i}\left(\lambda\right)\right)*\:{S}_{p,f,pixel}\left(\lambda\right)\:d\lambda\:$$

15 $$\:{P}_{b,pixel}\:=\:\int\limits_{{\lambda}_{i}}^{{\lambda}_{f}}\left(\prod\limits_{i=1}^{m}{f}_{i}\left(\lambda\right)\right)*\:{S}_{b,pixel}\left(\lambda\right)\:d\lambda\:$$

$\:{f}_{i}$ is each unique factor needed to describe physical interactions before the radiant power reaches the pixel, and m is the total number of these factors. These interactions can be a function of a variety of parameters depending on what interacts with the particle and background light before it reaches the image sensor. For the case of a color filter array or a similar arrangement of pixels that filter specific wavelengths, this filtering can be modeled through these factors. Additionally, the quantum efficiency, a wavelength dependent efficiency related to the pixels ability to convert photons to a charge, can be modeled through these factors^23^.

The quantity that a given pixel ultimately responds to is the total radiant energy collected over a sampling period, known as the exposure time. The radiant energy received is converted to a charge by the pixel, and this charge is the quantity that is processed to form the captured image^23^. The radiant energy collected for a given pixel in a set exposure time can be described by Eqs. (16)–(18).

16 $$\:{E}_{p,e,pixel}=\int\limits_{{t}_{0}}^{{t}_{e}}{P}_{p,e,pixel}\left(t\right)\:dt$$

17 $$\:{E}_{p,f,pixel}=\:\int\limits_{{t}_{0}}^{{t}_{e}}{P}_{p,f,pixel}\left(t\right)\:dt$$

18 $$\:{E}_{b,pixel}=\:\int\limits_{{t}_{0}}^{{t}_{e}}{P}_{b,pixel}\left(t\right)\:dt$$

$\:{E}_{p,e,pixel}$ is the radiant energy collected by a pixel from the particle elastic light scattering, $\:{E}_{p,f,pixel}$ is the radiant energy collected by a pixel from the particle induced fluorescence emission, $\:{E}_{b,pixel}$ is the radiant energy collected by a pixel from the background light sources, $\:{t}_{0}$ is the start of the exposure time, and $\:{t}_{e}$ is the end of the exposure time. The radiant power for each component must now be considered as time dependent as the particles may be moving or the incident light profile may be transient. The total radiant energy collected by the pixel during the exposure time can then be written as the summation of these three terms, assuming once again that they follow the superposition assumption.

19 $$\:{E}_{pixel}={E}_{p,e,pixel}+\:{E}_{p,f,pixel}+{E}_{b,pixel}$$

When evaluating the energy collected by a pixel and the overall particle signal, an important parameter to consider is the particle contribution compared to the background contribution. An effective way of quantifying this is the signal-to-noise ratio given by Eq. (20).

20 $$\:SNR=\:\frac{{E}_{p,e,pixel}+{E}_{p,f,pixel}}{{E}_{b,pixel}}$$

$\:SNR$ is the signal-to-noise ratio. The goal of a well-designed image detection sensor will be to maximize this signal-to-noise ratio. A high signal-to-noise ratio means that the pixels containing particles will be clearly distinguished from pixels only observing the background light sources. A high signal-to-noise ratio can be accomplished by having dark, non-reflective surfaces. It is also important to ensure that the amount of radiant energy collected from a particle, $\:{E}_{p,e,pixel}+\:{E}_{p,f,pixel}$, is greater than the detection threshold of a given pixel.

### Counting particle signals

The radiant energy collected at each pixel is converted by the image sensor to a pixel value. This conversion can vary depending on the image sensor; however, the result is a pixel value that is dependent on the amount of radiant energy collected during the exposure time. The gain of the image sensor, $\:{G}_{sensor}$, will dictate this conversion. The conversion can be represented generically by Eq. (21).

21 $$\:{I}_{pixel}={I}_{pixel}({E}_{pixel},{G}_{sensor})$$

Once an image is generated using the array of pixels and their associated values, the particle signals need to be differentiated from the background signal. In the simplest case, one can consider counting pixel by pixel. If the pixel value achieves a value greater than a set threshold, it can be flagged as a particle as described by Eq. (22).

22 $$\:{D}_{i}=\:\begin{array}{c}1,\:if\:{I}_{pixel}>\:{I}_{thres}\\\:0,\:if\:{I}_{pixel}<\:{I}_{thres}\end{array}$$

$\:{D}_{i}$ flags that a given zone, each pixel in this example, contains a particle, and $\:{I}_{thres}$ is the user-defined threshold. The threshold pixel value will be dependent on the signal-to-noise ratio given in Eq. (20). The total particle count for a given frame then could be found by examining each pixel and summing up each of the flags to get a total count, given in Eq. (23).

23 $$\:{N}_{count}=\:\sum\limits_{i=1}^{n}{D}_{i}$$

$\:{N}_{count}$ is the total number of particles counted for a given frame. This method of going pixel by pixel within the image would overestimate the particle counts as a particle signal is usually captured on more than one pixel. In general, any algorithm that can isolate the area of a continuous particle signal and define it as a single instance can be used. Since the detection algorithm will vary from sensor to sensor, it is best to rewrite Eq. (22) in more general terms.

24 $$\:{D}_{i}=\:\begin{array}{l}1,\:if\:detection\:criteria\:met\\\:0,\:otherwise\end{array}$$

With each of the particle zones flagged by the detection criteria, the number of particles within a given frame can then be counted using Eq. (23).

There are two important criteria to be considered for the number of particles counted per image. The first of these is the counting efficiency. The counting efficiency relative to the number of particles present in a captured image is defined by Eq. (25).

25 $$\:{\eta}_{counting}=\:\frac{{N}_{count}}{{N}_{total}}$$

$\:{\eta}_{counting}$ is the counting efficiency relative to the number of particles present in a captured image, and $\:{N}_{total}$ is the total number of particles in the image. For particle counting instruments, there is also a counting efficiency defined with respect to the total number of particles challenged by a sensor. The counting efficiency with respect to the particles challenged by the sensor is a function of many other parameters not covered here such as the fluid dynamics within the detection chamber. The focus of this framework is on the image detection method in a generic sense, so the counting efficiency is defined here based solely on the information contained within the captured images.

In addition to the counting efficiency, it is also important to quantify the number of false positives, areas of the image that meet the detection criteria due to noise or other factors but are not particle signals. This can be defined as a simple ratio between the number of false positives and the number of counted particles.

26 $$\:{\alpha}_{false}=\:\frac{{N}_{false}}{{N}_{count}}$$

$\:{\alpha}_{false}$ is the ratio of the false counts to the total particles counted, and $\:{N}_{false}$ is the number of false positives. A well-developed detection algorithm and a high signal-to-noise ratio are required to achieve a counting efficiency, $\:{\eta}_{counting}$, of unity and a small false count ratio, $\:{\alpha}_{false}$. By having a negligible false count ratio and a counting efficiency near unity, each particle within the image will be counted without any non-physical particles being counted.

### Differentiating bioparticle signals

For bioparticle detection, one must also consider the differentiation of bioparticles from abiotic particles. One way of accomplishing this is to apply a long-pass filter aimed at completely attenuating the elastic light scattering contribution. This method of isolating only the induced fluorescence emission mimics the approach of the previously mentioned commercial instruments and similar methods. Ideally, the filter attenuation factor for the elastic wavelengths would be equal to zero, leaving Eq. (27) to describe the radiant energy captured by a given pixel. The background light contribution is still included in this expression, but this contribution should also be greatly diminished.

27 $$\:{E}_{pixel}={E}_{p,f,pixel}+{E}_{b,pixel}$$

With a well-chosen filter, the particles that are counted will only be particles with a sufficiently strong induced fluorescence contribution. Equations (23)-(26) are still fully applicable for counting the number of bioparticles with this method. For Eq. (26) though, the number of false positives, $\:{N}_{false}$, should also include any abiotic particle signals that are detected due to leakage or other sources.

A second method of differentiation involves using the color of the particle signal to identify if an induced fluorescence contribution is present. Instead of aiming for complete attenuation of the elastic signal, the goal is to eliminate only the necessary amount of elastic light scattering until a color change due to the induced fluorescence contribution is observed.

Using this differentiation method, one can still get the total particle count per image using Eqs. (23) and (24). Another flag can be implemented that signifies whether a detected particle contains an induced fluorescence contribution or is purely elastic light scattering using the wavelength estimated by the color. An image sensor uses the previously mentioned color filter array, typically red-green-blue (RGB), and an interpolation process known as demosaicing to assign each pixel a corresponding color^23^. Assuming a typical RGB color filter array for example, a given pixel will be coated with a filter targeting a wavelength range defined for blue, a wavelength range defined for green, or a wavelength range defined for red. Each of these filters are described by their corresponding wavelength-dependent transmission curves^23^. As mentioned previously, this wavelength-dependent transmission of the radiant energy collected by a pixel can be implemented through the factors in Eqs. (13)–(15). Importantly, this means that the color assigned to a given pixel is a function of the radiant energy captured by itself and the differently filtered, neighboring pixels. This process of assigning a color to a given pixel can vary depending on the exact color filter array, and algorithm used by a given image sensor; however, if the generated image represents the color in some form, this method should be able to be adapted. Equation (28) describes this marker assuming only two channels are considered, fluorescence and no fluorescence. The color differentiation method could allow for many such channels to be considered, based on the observed color differences, without the need for complex sensor geometries.

28 $$\:{J}_{i}=\:\begin{array}{l}1,\:if\:{\lambda}_{calc}>\:{\lambda}_{cutoff}\\\:0,\:otherwise\end{array}$$

$\:{J}_{i}$ is a flag for an induced fluorescence signal, $\:{\lambda}_{calc}$ is the calculated wavelength of the signal based on the color, and $\:{\lambda}_{cutoff}$ is the cutoff wavelength defining whether a signal contains an induced fluorescence contribution or not. The number of bioparticles per frame can then be calculated using Eq. (29) and assuming that the induced fluorescence signals present are only due to biological sources.

29 $$\:{B}_{count}=\:\sum\limits_{i=1}^{n}{D}_{i}*{J}_{i}$$

$\:{B}_{count}$ is the number of bioparticles counted for the given frame. A separate counting efficiency can be defined for the counting of the bioparticles, given in Eq. (30).

30 $$\:{\eta}_{counting,bio}=\:\frac{{B}_{count}}{{B}_{total}}$$

$\:{\eta}_{counting,bio}$ is the counting efficiency relative to the number of bioparticles present in the image, and $\:{B}_{total}$ is the total number of bioparticles within the image. Similarly, the number of false positives can be quantified for the bioparticle counting, given by Eq. (31).

31 $$\:{\alpha}_{false,bio}=\:\frac{{B}_{false}}{{B}_{count}}$$

$\:{\alpha}_{false,bio}$ is the ratio of the false positives to the total bioparticles counted, and $\:{B}_{false}$ is the number of false positives. The bioparticle false positives will include both abiotic particle signals and noise signals incorrectly identified as bioparticles. This method requires consideration of Eqs. (23)–(26) and Eqs. (28)–(31) to obtain both accurate counting of the total particles present and accurate differentiation of bioparticles from abiotic particles.

### Results and discussion

#### General image detection

**Fig. 2:**
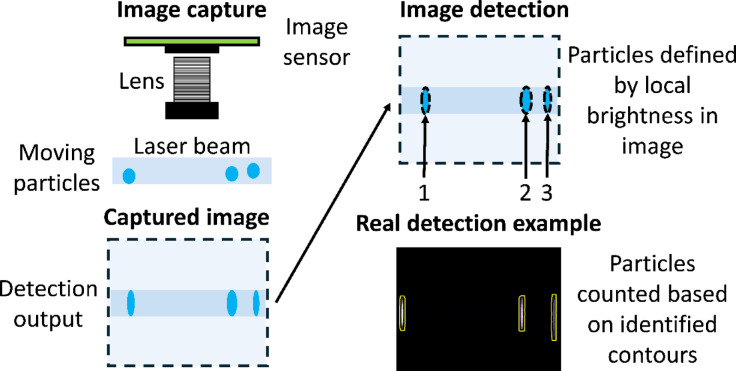
Diagram of the general detection method used by the prototype sensor in this evaluation. One or more optical filters can be placed between the image sensor and the lens to attenuate a wavelength range of interest.

A sensor was developed to utilize image detection of particles and validate the proposed framework. The general detection method is given below in Fig. 2.

The particles pass through a collimated laser beam oriented horizontally across the image. The elastically scattered light and the emitted induced fluorescence light is then collected by the image sensor, which generates a corresponding image. The particle signals are then isolated from the background signals using the procedure described in the methods section. The individual particle signals are counted, yielding a total particle count for that frame. In this way, the image detection method acts as multiple instances of single particle counting for each of the isolated signals within a captured image. This removes some of the ambiguity associated with other optical counting methods since it is possible to directly view each of the individual particle signals, as opposed to relying upon an electrical pulse to detect the presence of a particle.

**Fig. 3:**
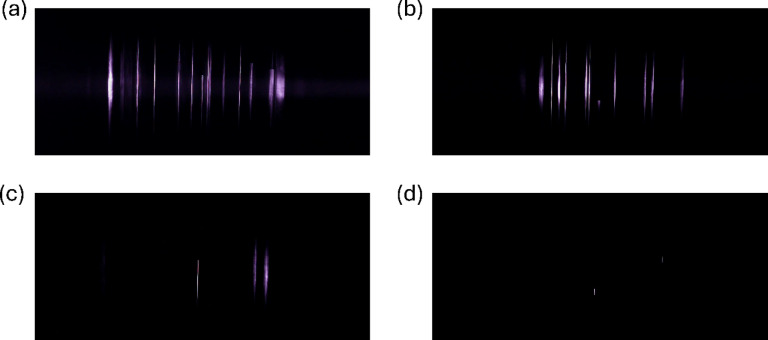
Captured images for 2.87-µm NaCl particles passing through a 375-nm laser with no filter applied. All images were taken with particle concentrations at approximately 40 #/cm^3^. Note that the images are cropped to focus on the illuminated area. (**a**) Exposure control value set to -2. (**b**) Exposure control value set to -4. (**c**) Exposure control value set to -6. (**d**) Exposure control value set to -10.

As covered in Eqs. (16)–(18), one of the key parameters that must be set is the exposure time. In the case of the image sensor used here, the exposure time is selected via an exposure control value ranging from 0 to -13. From the camera manufacturer, the exposure control values of -2, -4, -6, and -10 correspond to exposure times of 250 ms, 62.5 ms, 15.6 ms, and 0.98 ms respectively; however, these exposure times were not verified and should be taken as estimations. Importantly, the larger the value, in this case the closer to zero, the longer the exposure time. The effect of changing the exposure time is shown in Fig. 3.

Figure 3a contains the largest number of particles out of the four different exposure times. There is also a notable increase in the scattering from the medium within the laser path for Fig. 3a. As the exposure time is increased, more particle signals are present as there is more time for particles to pass through the illuminated region. The background signal also grows during this extended time due to the contribution from the medium and background. A shorter exposure time means that particles will not have sufficient time to pass through the illuminated area, so fewer particle signals are captured and those that are captured can be incomplete if the exposure time is set too short, as seen in Fig. 3d. The benefit to this shorter exposure time is the decrease in the background brightness level.

With the dark background observed here and the ability to easily distinguish the particle signals from the background, even at an exposure control value of -2, there would be no clear reason to set the exposure control value to -6 or -10 in relation to the image quality. The potential benefit to these shorter exposure times would be being able to operate the image sensor at a higher frame rate. Operating at a higher frame rate reduces the chances of saturating the viewing area but requires increased computational power to apply the detection algorithm at the increased pace. The effect of changing the exposure time on the average particle counts is shown quantitively in Supplemental Fig. S1.

The trends observed for this sensor design are a departure from those expected for stationary particles. While the framework allows for both moving particles and stationary particles to be described, the prototype sensor specifically focuses on airborne, moving particles. Since the moving particles can pass through the viewing area within the exposure time, increasing the exposure time beyond a certain limit will not yield an improvement for a given signal’s brightness. For stationary particles, increasing the exposure time will allow for the particles to contribute more radiant energy to a given pixel as they will be scattering and emitting light during the full duration of the exposure time, assuming negligible saturation of the pixels. This allows for stronger particle signals simply by adjusting the exposure time. This was shown by Swanson and Huffman through varying the exposure time of deposited 2.0-µm fluorescent PSL spheres^13^. This represents a significant trade-off between the method employed by the prototype sensor described here and other similar detection methods that focus on stationary particles.

**Fig. 4:**
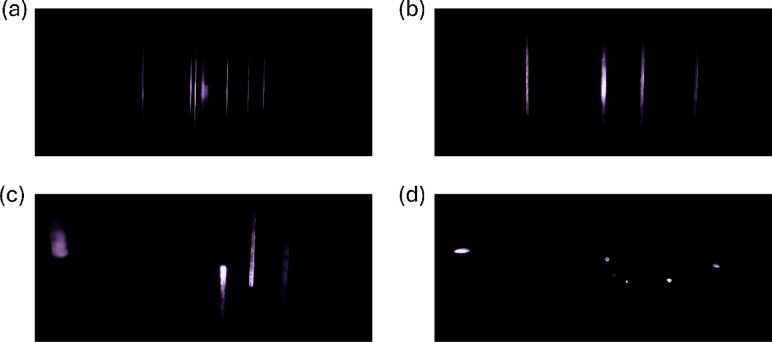
Captured images for 2.87-µm NaCl particles passing through a 375-nm laser with no filter applied and a constant exposure time between cases. It is assumed that the average flow speed reflects that of the average particle speed; however, deviations may be present due to inertial effects. Note that the images are cropped to focus on the illuminated area. (**a**) Average flow speed of 1.0 m/s. (**b**) Average flow speed of 0.6 m/s. (**c**) Average flow speed of 0.1 m/s. (**d**) Particles captured after stagnating the inlet flow.

Contributing in a similar manner to the particle signals is the particle speed through the illuminated area. Figure 4 depicts images of particles captured when moving through the sensor at three different average flow velocities and one image of stagnated particles.

In Figs. 4a−c, the moving particle signals have a streak-like geometry. The faster particles will only contribute a notable amount of light to a given pixel for a short amount of the exposure time. Once past the region of a given pixel, the contribution from the particle will be near zero, so the overall light collected from the particle, $\:{E}_{p,e,pixel}+\:{E}_{p,f,pixel}$, will be less for a given pixel than for a slow-moving or static particle. At the edges of the particle signal where the pixel value is usually the least, this increase in the particle speed will cause the edges to be less distinguishable from the background. For Fig. 4c, the particles are moving slow enough that a clear termination of the signal is observed inside the illuminated area. Figure 4d displays that stagnated particles will appear bright and confined compared to the other cases due to the limited motion during the exposure time. By stagnating the particles, it may be possible to replicate the behavior of stationary particles, where a continuous benefit can be obtained from increasing the exposure time.

Using the process described in the methods section, the SNR as a function of particle speed can be estimated. The SNR with 95% confidence was estimated to be 16,000 ± 800, 330 ± 20, 166 ± 7, and 127 ± 4 for the stagnant case, the 0.1-m/s case, the 0.6-m/s case, and the 1-m/s case respectively. Decreasing the average flow speed through the camera viewing area results in a higher SNR. A nearly two orders of magnitude jump is observed between the 0.1-m/s case and the stagnant particle case. This is due to the issue of fewer missed particle signals for the stagnant particle case, resulting in a significantly lower background value. This likely inflates this metric for the stagnant particle case. Comparing just the average pixel value, the stagnant case has an average of 71 ± 1.5, the 0.1-m/s case has an average of 63 ± 2, the 0.6-m/s case has an average of 54 ± 1.0, and the 1-m/s case has an average of 46 ± 0.7. This further reinforces the observed trend that decreasing the flow speed increases the SNR, without consideration of the missed particle signals. It is important to mention that the 0.1-m/s particles may not fully cross the laser profile as shown in Fig. 4c. This could be inflating the average value as the contour captured will not feature the final portion of the particle signal where the pixel value decreases again due to the change in the laser profile.

While the signal quality may be negatively impacted by faster particle speeds, the faster the particles and the flow move through the image area, the more particles that can pass through the image during the exposure time. This leads to a competing effect of more particles being present in each image, but potentially a worse counting efficiency due to the diminished particle signal. The effect of this increased particle speed on the average particle count per frame is displayed in Supplemental Fig. S2.

Based on the results from the prototype sensor, the velocity threshold where the particle signals worsen notably is approximately 1 m/s for the elastic scattering. With an inlet diameter of 3.2 mm, this corresponds to an inlet volumetric flow rate of 0.5 L/min, which is comparable in magnitude to the 0.3-L/min sample flow rate of the WIBS^6^. Currently though, the prototype sensor is not focusing the particles through the laser profile. This lack of focusing means that not every particle is challenged equally. Because of this, the prototype sensor likely challenges less particles than the WIBS despite the similar flow rates. This will require further improvement and optimization as the image sensor allows for particles to be spatially distributed in the viewing plane, but confinement in the out-of-plane direction is still desired to ensure each particle is being challenged (i.e. not out of focus or removed from the peak laser power).

**Fig. 5:**
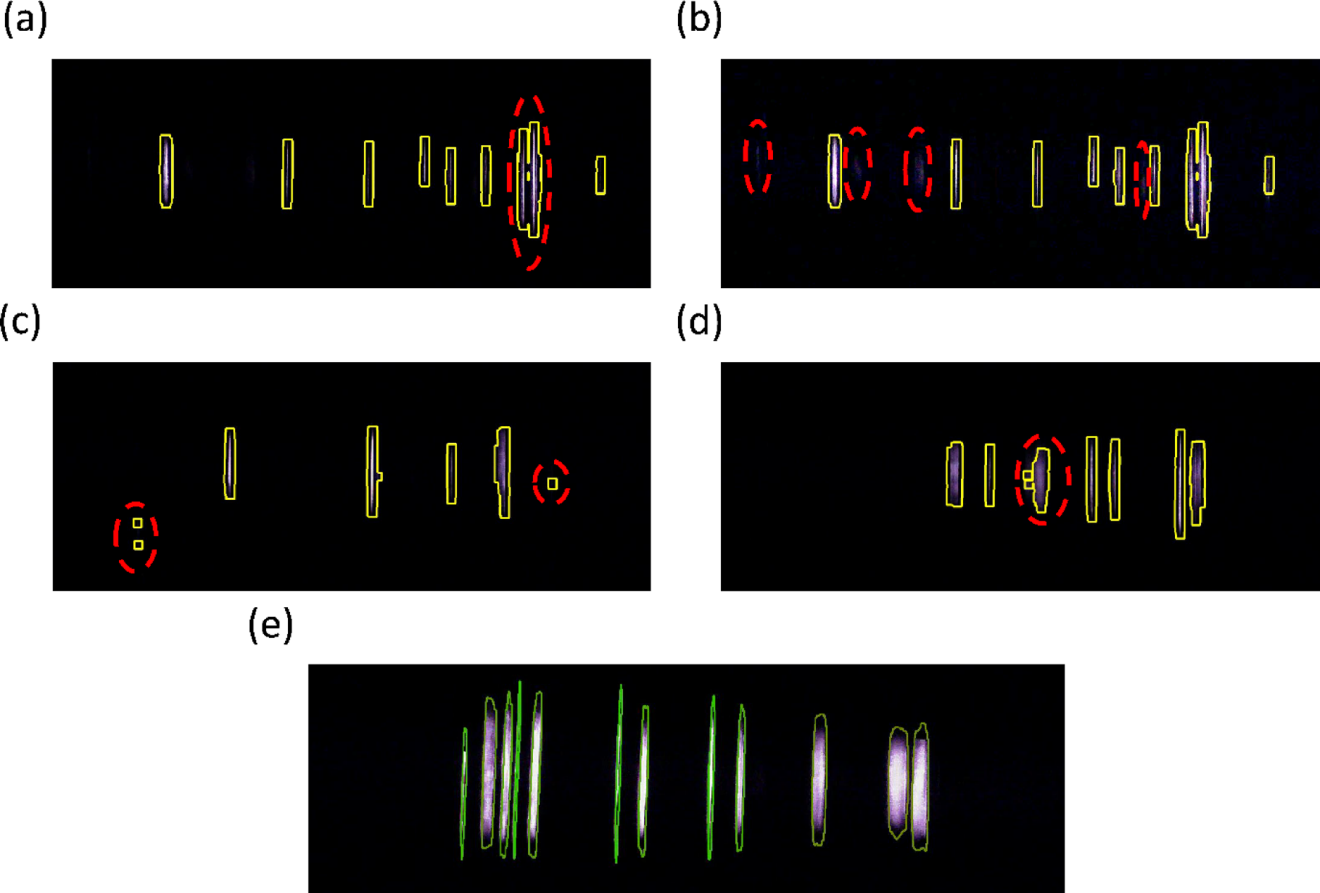
Isolated screenshots of elastic scattering from 2.87-µm particles illuminated with a 375-nm laser and with no filter applied. Note that the images are cropped to focus on the illuminated area. (**a**) Screenshot showing two particle signals merged into a single particle signal. (**b**) A brightened and higher contrast version of (**a**) showing the visible particles missed by the detection algorithm. (**c**) Example showing unclear particle signals away from the main area of illumination. (**d**) Example showing a single signal being counted as multiple particles. (**e**) Example showing the updated detection algorithm tailored to the moving particle signal geometry.

The movement of the particles is just one such consideration that must be made when designing an effective particle detection algorithm. There are many factors that must be considered to get a counting efficiency, $\:{\eta}_{counting}$, near unity and limit the false count ratio, $\:{\alpha}_{false}$, to an adequately low level. Figure 5 depicts a few of the common issues encountered with having a simple detection algorithm and an updated detection algorithm to address these issues.

In Fig. 5a, two clear particle signals are observed, but the detection algorithm interprets the two signals as one. A robust detection algorithm will need to be able to handle cases where the contours are either close to or are overlapping one another. Without this issue fully addressed, the summation of the particle flags in Eq. (23) will yield a lower total than the image contains. This will decrease the counting efficiency, $\:{\eta}_{counting}$.

In Fig. 5b, it is possible to identify 4 different areas where faint particle signals are present. As mentioned in the framework, achieving a high signal-to-noise ratio, SNR, will allow these fainter signals to be detected without having to worry about an excessive number of false positives. In these images, the signal-to-noise ratio appears high, allowing for these faint signals to be counted if the detection algorithm was tuned further.

If the detection algorithm is too aggressive, it is possible to encounter the issue in Fig. 5c. In Fig. 5c, contours are applied to regions of the image that do not have clearly visible particles, resulting in an increased total particle count. It is a balance to count the faint particle signals like those in Fig. 5b while avoiding counting noise such as those in Fig. 5c. These two cases will need to be balanced to achieve a counting efficiency, $\:{\eta}_{counting}$, near unity and limit the false count ratio, $\:{\alpha}_{false}$, to an adequately low level.

The final issue, shown in Fig. 5d, is a single particle signal being counted as three different particle signals. This is especially common for blurry signals caused by out-of-focus particles. These blurry signals can have areas of high and low intensity, leading to multiple separate particle signals being detected despite it only being one true particle signal. Proper focusing of the optics can help mitigate this issue.

For the preliminary detection algorithm used, shown in Figs. 5a-d, a high sensitivity to small signals was required to count the focused particle signals, resulting in many false positives. For accurate quantification of particles, a more sophisticated detection program is required to supress the high number of false counts while maintaining a counting efficiency near unity. This is achieved by developing the detection algorithm based on the streak-like geometry of the moving particle signals rather than just general contours. An example of such a detection algorithm is shown in Fig. 5e and described in the methods section. The updated detection algorithm is more effective at detecting the thin, dull signals without introducing many false counts.

For the preliminary detection algorithm, the false count ratio was approximately 14% with an average counting efficiency of 1.0. This was estimated using the process described in the methods section. The combination of these two metrics displays that the preliminary detection algorithm had a substantial number of false positives but also missed a considerable number of visible signals as well. The updated code performs much better with a false count ratio of approximately 3.5% and an average counting efficiency of 1.0. This displays that there are still false counts that can be reduced and particle signals that are not being counted, but it is within an acceptable level, especially considering overlapping particle signals. It is important to note that these metrics are specifically for the elastic scattering detection of 2.87-µm NaCl particles at an average flow speed of 0.6 m/s and an exposure control value of -4. These metrics, especially the counting efficiency, are dependent on the signal strength and operating conditions, so the reported metrics should be considered as under optimal conditions. Further improvements can still be made to improve these metrics and the overall detection quality, especially for weak particle signals.

#### Induced fluorescence detection: removal of elastic light scattering

**Fig. 6:**
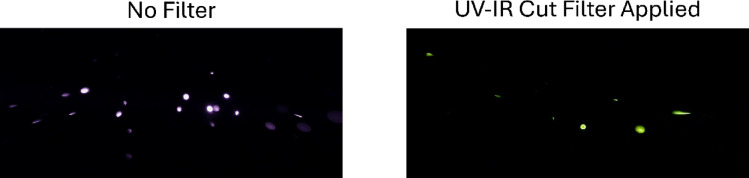
Isolated images of pure riboflavin particles illuminated with a 375-nm, 300-mW laser with and without a UV-IR cut filter (Gzikai) applied in line with the image sensor. The images are of an unknown size and concentration of particles generated through nebulization. The images were captured after stagnating the particle flow. Note that the image is cropped to focus on the illuminated area. The UV-IR cut filter has a reported transmission of 93% at a wavelength of 415 nm.

The previous discussion focused on the general detection of particles using the image detection method; however, bioparticle detection has its own considerations. Figure 6 shows an example of the method described where only the induced fluorescence contribution is visible through the application of a UV-IR cut filter (Gzikai), which has a reported transmittance of 93% at 415 nm.

Without a filter applied, the image appears almost identical to the NaCl images shown in the previous figures, specifically that of Fig. 4d. The reason for this similarity is that the dominant term for the light collected during the exposure time is $\:{E}_{p,e,pixel}$. Even though there may be a contribution from the induced fluorescence emission, this contribution, $\:{E}_{p,f,pixel}$, is much smaller in magnitude than the elastic light scattering contribution. When viewing the particle signals without a filter, the captured signals will reflect this dominant, incident wavelength, hence the violet color assigned to the particle signals.

Riboflavin is expected to have induced fluorescence emission in the range of 500 to 600 nm for an excitation wavelength near the 375-nm laser^24^. This higher wavelength emission is the reason that the color of the filtered riboflavin particles is more green-shifted/red-shifted than the elastic scattering, as this wavelength range is expected to be detected more heavily by the green and red-designated pixels of the color filter array^23^. This also signifies that the bulk of the riboflavin induced fluorescence emission is removed from the wavelength of the incident light. This allows for a filter that attenuates the elastic light scattering without having to attenuate the induced fluorescence emission. Additionally, the incident wavelength used here is well-suited for the excitation of riboflavin, so the fluorescence cross-section is expected to be significant. This large fluorescence cross-section results in a high intensity signal for the riboflavin, depicted by the large and bright signals in Fig. 6.

Currently, a filter and excitation wavelength combination that fully removes the elastic scattering and still allows for consistent counting of the induced fluorescence signals has not been achieved. The particle counts of 1% mass riboflavin particles at two different diameters are given in Supplemental Fig. S3 and Supplemental Fig. S4. For 2.87-µm particles, counts of approximately 60% of the elastic scattering counts for the same conditions were achieved. This displays that the counting is nearing the required limit for accurate quantification for these particles but has not yet reached it. Moving to 1.33-µm particles of the same composition leads to essentially zero particles detected per frame regardless of the input particle concentration. This displays that the current sensitivity and particle illumination is insufficient to properly detect particles of this size and composition. Further optimization of the particle illumination and sensor sensitivity will be required to achieve consistent quantification.

While the 1% mass riboflavin detection shows the potential of the method and prototype sensor, the sensor still requires significant optimization and improvement in terms of sensitivity before it is expected to yield accurate bioaerosol detection and quantification. The chemical composition of *Bacillus* vegetative cells chronicled by Hill et al. 2015 lists a dry weight mass fraction of 0.007% flavin mononucleotide + riboflavin^22^. This gives an idea of the mass percentage of riboflavin expected in real bioparticles. To consistently detect a 3-µm *Bacillus* cell, a roughly 2 orders of magnitude improvement in sensitivity would be required when considering this fluorophore by itself and assuming the signal strength is proportional to the mass of the fluorophore present. When considering the combination of this fluorophore and other fluorophores present in a bioaerosol, such as NADH which has a listed dry weight mass fraction of 0.062% (combined with NADPH) and tryptophan which has a listed dry weight mass fraction of 4%, the improvement required is expected to be less^22^. The difficulty will be exciting all the fluorophores present. Detection of smaller particles will require further improvements to the sensitivity. With the current sensor design, it is expected that the detection limit will be 1-µm or larger bioparticles; however, verification of this limit will need to be investigated.

#### Induced fluorescence detection: color differentiation

**Fig. 7:**
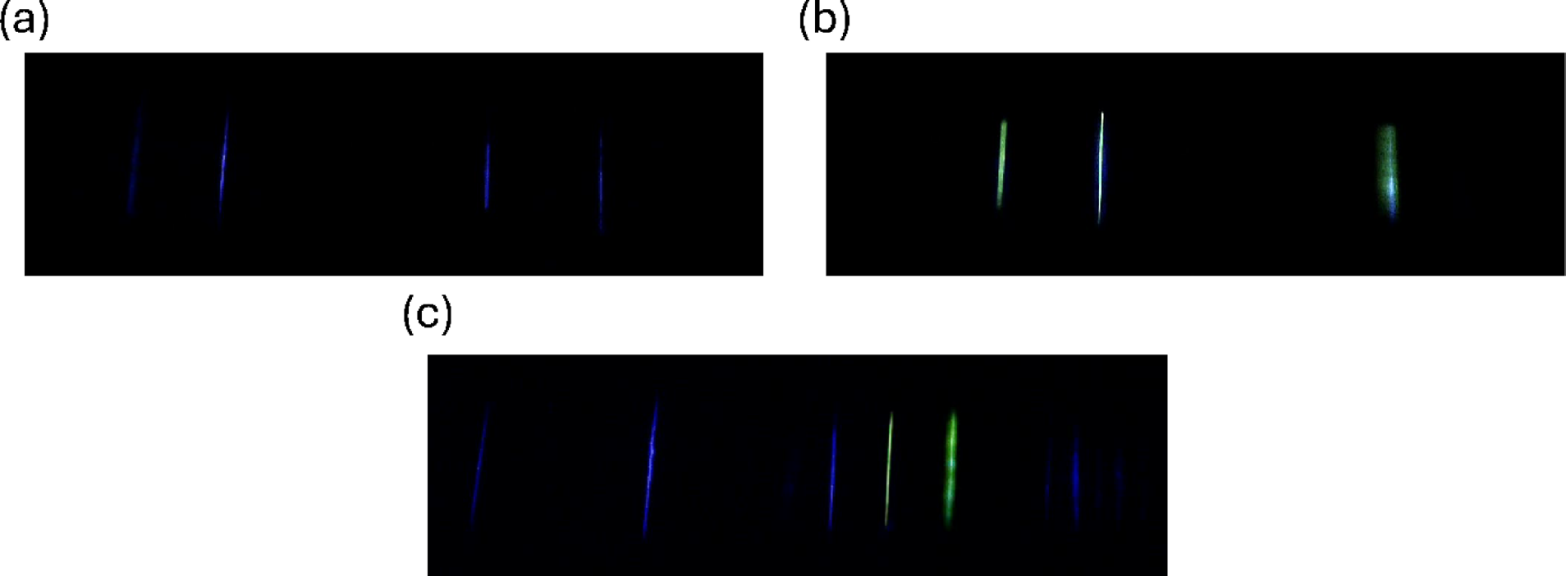
Isolated images depicting the color difference due to induced fluorescence contributions for particles illuminated with a 405-nm, 300-mW laser. Note that the images are cropped to focus on the illuminated area. (**a**) 2.87-µm NaCl particles with two UV-IR cut filters (Gzikai) applied, an exposure control value of -4, and a particle speed of 0.1 m/s. (**b**) 2.87-µm 1% mass riboflavin particles with two UV-IR cut filters (Gzikai) applied, an exposure control value of -4, and a particle speed of 0.1 m/s. (**c**) An unknown concentration of NaCl and 1% mass riboflavin particles with two UV-IR cut filters (Gzikai) applied. The UV-IR cut filters have a reported transmission of 93% at a wavelength of 415 nm.

The second method of differentiation proposed in this framework involves using the particle signal color to identify an induced fluorescence contribution. Figure 7 depicts the implementation of this method for NaCl, and 1% mass riboflavin particles using the combination of two UV-IR cut filters (Gzikai) and a 405-nm laser.

A visible color difference is observed when comparing the NaCl and 1% mass riboflavin signals in Fig. 7a and b. As was mentioned previously, the color of the observed signals is a function of the radiant energy collected by a given pixel compared to the neighboring pixels. The NaCl particles will mainly contribute to the pixels focused on the lower wavelengths, particularly the blue-designated pixels, due to only elastically scattering the incident 405-nm light. This results in the color of these signals being designated as the blue color observed in Fig. 7a. For the 1% mass riboflavin particles, they have a significant contribution to the pixels focused on the higher wavelengths, particularly the green-designated and red-designated pixels, from the induced fluorescence emission. While the elastic scattering contribution should be similar to the NaCl particles, the increased contribution to the green-designated and red-designated pixels results in a more green-shifted color being assigned to the 1% mass riboflavin signals. The exact color can vary between individual signals as shown in Fig. 7b, depending on the signal quality and potentially slight changes in the particle composition. As is observed in Fig. 7c, the mixture of 1% mass riboflavin and NaCl particles shows two distinct colors, showing the potential to differentiate biological and abiotic signals simultaneously.

To further illustrate the color difference between the signals, the hue of the detected contours, a representation of the color described by the angle on a color wheel, was calculated for the particle compositions shown in Fig. 7a and b. This was completed for six different detection videos of each particle type, a total of 7200 frames. The average hue for each particle type was calculated by averaging the hue of all contours detected within these frames. The average hue with 95% confidence was found to be 224° +/− 1° and 142° +/− 5° for NaCl and 1% mass riboflavin, respectively. This further highlights the observed color differences in Fig. 7a and b, and that it can be represented quantitively. For example, the hue could be used to differentiate the presence of an induced fluorescence contribution by correlating this metric to an estimated dominate wavelength and then setting $\:{\lambda}_{cutoff}$ in Eq. (28) to the wavelength estimated for a hue of 200°. Using this hue as a cutoff for the previously mentioned detection videos, the riboflavin particles would be identified as particles with an induced fluorescence contribution with 99.3% accuracy; however, approximately 10% of NaCl signals would also be identified as particles with an induced fluorescence contribution as well. Further work will be required to limit these deviations and keep signals from being misidentified.

The counting of the particle compositions given in Fig. 7a and b with two UV-IR cut filters (Gzikai) applied and illuminated with a 300-mW, 405-nm laser are given in Supplemental Fig. S5. At a diameter of 2.87 μm, the NaCl particles are counted at roughly 50% of the level that the 1% mass riboflavin particles are being counted at with this setup. This displays that the elastic scattering attenuation is too significant with this setup to achieve a counting efficiency of unity for the total particle counts, as particles with no induced fluorescence contribution at this size will be counted at a reduced rate. This represents one of the main struggles with this implementation. A filter will need to be chosen that does not affect the induced fluorescence emission wavelengths and only attenuates the elastic light scattering wavelengths to the required level. The required attenuation could vary depending on the incident light level and the particle composition of interest. It is still not fully evaluated what level of elastic scattering detection can be achieved while maintaining color differences like the ones displayed here.

Pertaining to the differentiation between different fluorophores, this aspect of the method is expected to be limited by the wavelength difference between the induced fluorescence emissions and the accuracy that the color can be assigned. For tryptophan and riboflavin as an example, there is expected to be a notable difference between the two emission wavelength ranges^24^. This allows for more room for the two fluorophore signals to deviate in color while still being accurately differentiated. These deviations can be from differences in the individual particle compositions and noise associated with the radiant energy collected by a given pixel. For fluorophores with a similar emission range, these deviations in the color may compromise the ability to consistently differentiate two different fluorophores. Another challenge will be managing differences in the elastic scattering contribution. If a particle has a different ratio of an elastic scattering contribution to the induced fluorescence contribution even when containing the same fluorophore, the signal color would skew further or closer to the incident wavelength color. This may limit the ability to differentiate different fluorophores from one another for particles with significantly different sizes or optical properties.

## Conclusion

The proposed framework effectively outlines the process of using an image sensor for the detection of bioaerosols using the induced fluorescence emission from such particles. From the interaction of both abiotic and biological particles with a given incident light source to the implementation of a detection algorithm to quantify the corresponding signals, each step of the interaction is mathematically described to highlight the importance and the required optimizations associated with each step of the detection process. While the assumptions and simplifications made in the derivation of this framework may need further expansion and consideration before exact quantitative values can be obtained, it is believed that this framework provides a valuable tool for those looking to implement image detection of bioaerosols in a novel manner or refine existing instances of such methods. Further expansion of the framework could allow for complex interactions, such as the interference between the medium and particle scattering, to be considered.

As far as the prototype sensor itself, the experimental evaluation displayed that an updated detection algorithm, tailored to the signal geometry, could achieve a false count ratio of approximately 3.5% and a counting efficiency near unity at optimal detection conditions. Additionally, the evaluation displayed that a compromise must be made when controlling the particle speed through the detection region. The faster particle speeds contributed to a reduced SNR but allowed for an increased number of particles to appear in each image. Similarly, it was shown that the exposure time must be selected to give enough time for the particle signals to be well-captured, but short enough to avoid saturation of the detection region and excessive background brightness. These trends can all be described by the proposed framework, and lend credence to the accuracy of the framework, despite the simplifications and assumptions implemented.

Pertaining to the bioaerosol detection capabilities, evaluation of 1% mass riboflavin particles validated that applying a long-pass filter to remove all elastic scattering can be used to differentiate and detect induced fluorescence signals with this setup; however, the detection of this fluorophore will need significant optimization before the sensor can be applied to real bioaerosol detection. To detect real bioaerosols near 1 μm in diameter using this method, an estimated two orders of magnitude improvement will be required. The second proposed method of differentiation, using the color difference between the elastic scattering contribution and the induced fluorescence contribution, was shown to be a plausible approach, both in the framework and experimentally. A clear difference in the average hue, approximately 82°, was observed between filtered NaCl signals and filtered 1% mass riboflavin signals. Use of this difference would allow for approximately 99.3% of 2.87-µm 1% mass riboflavin particles to be identified as having an induced fluorescence contribution; however, 10% of 2.87-µm NaCl particles would also be identified as such. More work will be required to validate whether the balance between having a clear color difference and preserving the required elastic light scattering contribution can be achieved.

Future work will be focused on further developing the image-based bioaerosol sensor, using this framework for guidance. Classification of the laser profile, excitation parameters, and other key factors will allow for a more quantitative evaluation of the framework and analytical optimization of the prototype sensor. Outside of the interactions described by the proposed framework, additional work is required to optimize the sensor fluid dynamics and the particle counting efficiency in terms of the total number of particles challenged by the sensor. This introduces additional parameters that must be addressed in tandem with the parameters identified in this framework.

### Methods

**Fig. 8:**
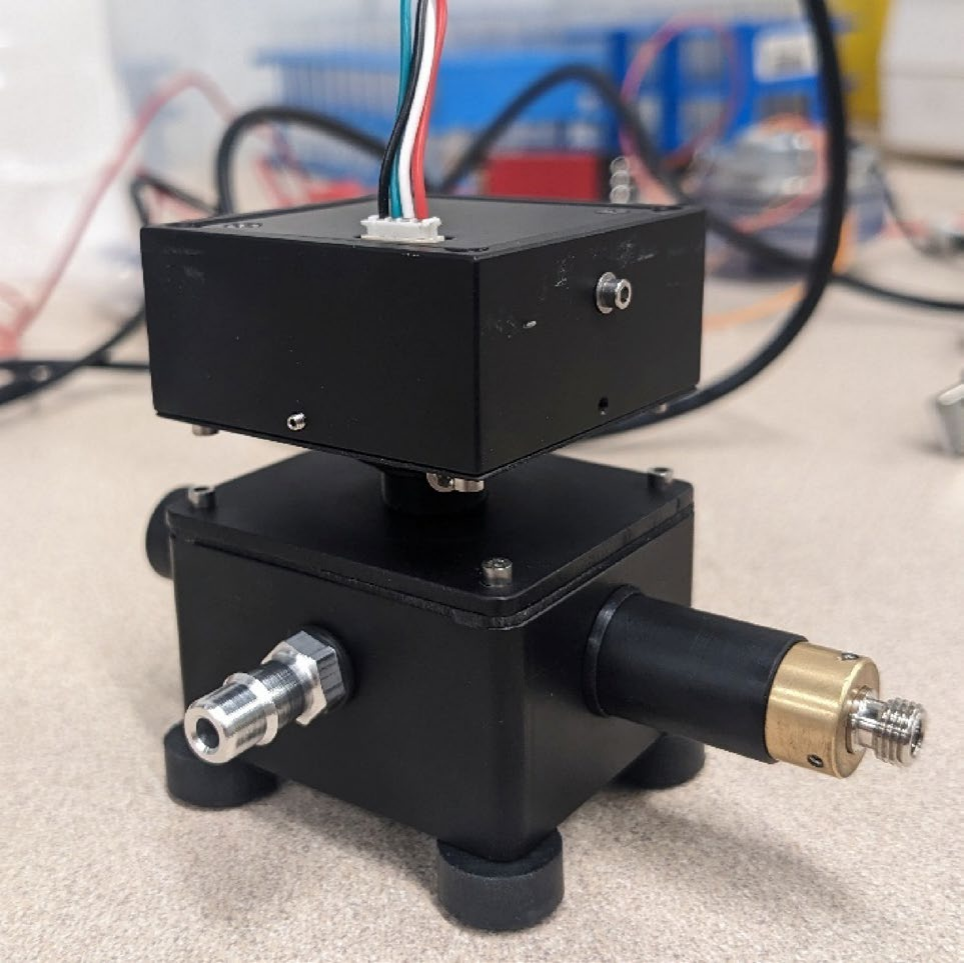
Photograph of the prototype sensor used to investigate the image detection method. The lower module is the main detection chamber. This module contains the inlet and outlet ports, a connector for the laser, a laser dump, and a thread for the imaging lens. The surfaces of the detection chamber are matte-black anodized aluminum to reduce background light sources. The rectangular module on the upper portion of the sensor is a custom focusing mechanism that allows for the distance from the lens to the image sensor and the orientation of the image sensor to be adjusted independently.

A photograph of the sensor used to evaluate the proposed framework is given below in Fig. 8.

A 405-nm 300-mW laser and a 375-nm 300-mW laser were used for the evaluation. The input power to the lasers was controlled with adjustable power supplies. The laser beam was focused using a collimating lens. The sensor chamber featured a 3.2-mm circular inlet aligned with the laser beam such that each particle passed at least once through the laser beam. A 6-mm, 5-MP, wide angle lens was positioned above the focused beam to observe the particles passing through the illuminated area. An image sensor (i.e. camera), Sony IMX291, was positioned such that it looked down at the illuminated area and the region surrounding it. The image sensor position was adjusted using a custom focusing mechanism, such that well-focused images of the particles passing through the laser beam were captured. The focusing mechanism allows the distance from the lens to the image sensor and the orientation of the image sensor to be adjusted independently, a necessary feature for optimal focusing and consistent quantification. Timed samples of the images were collected using a custom-Python software, allowing for the camera settings, detection settings, and analysis settings to be adjusted manually. The quantitative results given in Supplemental Figs. S1 – S2 and Supplemental Fig. S5 were captured at a frame rate of 15 FPS. The results in Supplemental Figs. S3 and S4 were captured at a frame rate of approximately 7.5 FPS.

To mimic the detection of micron-sized bioaerosols, a Flow Focusing Monodisperse Aerosol Generator (TSI Model 1520, FMAG) was used to generate solid particles of varying diameters. The particle diameters reported in the evaluation are geometric particle diameters. Through the variation of the particle solution concentration, droplet generation frequency, dilution air flow rate, and solution liquid flow rate, the size and concentration of the generated particles was controlled^25^. Solutions ranging from 0.001 to 0.0001 volume fractions were required for the generation of particles in the size range of 1 μm to 3 μm. Supplemental Table S3 outlines the parameters required for the generation of each particle diameter tested. NaCl particles were generated to observe purely elastic light scattering as there should not be an induced fluorescence contribution from such particles. Riboflavin was chosen as the evaluated fluorophore as it is commonly cited as an indicator for the presence of bioaerosols and has an excitation wavelength within the desired range^22,24^. All solutions were prepared using HPLC-grade water. The riboflavin was mixed with NaCl for the quantitative evaluation depicted in Supplemental Figs. S3–S5 because of the difficulty reaching the required solution concentrations and to better mimic real bioparticles.

To serve as a reference for the particle size distribution and the number concentration of the generated particles, an Aerodynamic Particle Sizer (TSI Model 3321, APS) was used in parallel with the prototype image sensor. The Aerodynamic Particle Sizer recorded both the particle size distribution and the average number concentration of the generated particles during the sampling period. By recording this, the number of particles counted during the sampling period with the prototype sensor could be directly correlated to a given particle number concentration. To ensure a true monodisperse distribution of particles, a multiplet reduction impactor was placed downstream of the FMAG^26^. The volumetric flow rate through the prototype sensor was controlled using a calibrated mass flow controller controlled via a LabVIEW program. The flow rate was recorded at a rate of 1 Hz with the data being written to a .CSV file for future reference.

To capture the image in Fig. 6, a nebulizer was used to disperse a high concentration of pure riboflavin particles. For this generation method, the size and the concentration of the particles generated were unknown. The results of these nebulized particles were not used in quantitative analysis and were specifically used for illustrative purposes.

For the preliminary detection algorithm used to identify the particle signals shown in Figs. 5a-d, the image was first converted to grayscale. After this conversion, a blur was applied to reduce the level of noise in the image. Next, OpenCV adaptative thresholding was used to process the captured images and identify pixels that could be considered part of a particle signal. The OpenCV adaptive thresholding assigns a high value, 255, or a low value, 0, to the pixels above an adaptively defined threshold based on the local area around the pixel. After completing this thresholding, the OpenCV FindContours function was then used on this processed image. The OpenCV FindContours function uses an algorithm to determine contours based on the borders of the high value and low value pixels^27^. The number of particles within the captured image was then defined as the number of independent contours. The number of particles counted for each image was then saved to a .CSV file for future analysis.

For the updated detection algorithm used to collect the quantitative data in Supplemental Figs. S1–S5 and shown in Fig. 5e, an algorithm specifically focused on the streak-like geometry of the moving particle signals was employed. The captured images were converted to a gray-scale image, and a vertical blur was applied. Once the blur was applied, two separate adaptative thresholds were applied to the image. The first of these was focused on identifying blurry, out-of-focus particle signals. Only these types of signals were kept based on the calculated aspect ratio. The second adaptive threshold was focused on identifying the particle signals that are long and narrow. The results from both adaptive thresholds were combined into a single image. Small signals, those associated with noise, were then removed from the combined image. The remaining signals were then connected in the vertical direction as the images periodically featured disconnected zones for a given particle signal. Finally, the OpenCV FindContours function was used to identify the number of particles within the captured image. This particle count was then saved to .CSV file for analysis.

To determine the average pixel value and the SNR, the updated detection algorithm was used without the vertical blur applied. The average pixel value for each identified contour was found using the grayscale image, with a maximum value being 255. The individual contour values were then averaged to yield an average pixel value for the given detection video. For each frame, the average background pixel value was calculated by averaging the pixel value of all pixels not included in the contours identified as particle signals. This was done for detection videos zoomed into the particle signals to avoid including the background far from the detection region. These were then averaged for all frames of the detection video. The SNR was then calculated using Eq. (20), assuming that the pixel value was proportional to the radiant energy collected by each pixel.

To estimate the false count ratio and the counting efficiency of the detection code, the number of particles the detection code counted for each frame was compared to the visual counting of distinct particle signals within the image for 100 frames. Additionally, the number of false counts per frame was also quantified. This was done for an exposure control value of -4 and an average flow speed of 0.6 m/s at three different concentrations. The counting efficiency of the detection code and the false count ratio were calculated for each frame and averaged across the 100 frames, using Eqs. (25) and (26). Evaluating both metrics relies upon user judgement, so these serve mainly as rough estimations of these parameters.

## Supplementary Information

Below is the link to the electronic supplementary material.

Supplementary Material 1

## Data Availability

Data available on request from the corresponding author.
