# Supplementary material for: A framework for real-time image detection of bioaerosols
Source: Sci Rep. 2025 Dec 29;16:2827. doi: 10.1038/s41598-025-32744-x (PMC12824191; doi:10.1038/s41598-025-32744-x)
Supplement: Supplementary file 1 — Supplementary Material 1 [file 41598_2025_32744_MOESM1_ESM.pdf]

Supplementary Materials:

A Framework for Real-Time Image Detection of Bioaerosols

Authors: Ryne A. Juidici[1*], Yan Ye[1,2], Francisco J. Romay[1], Miles Owen[3], Qisheng Ou[1], David Y.H. Pui[1*]

[1]Mechanical Engineering, University of Minnesota, Minneapolis, Minnesota, United States.

[2]Y2Y Technology, Santa Clara, California, United States.

[3]U.S. Army Primary Standards Laboratory, Redstone Arsenal, Alabama, United States.

Corresponding Authors: Ryne A. Juidici, juidi002@umn.edu, David Y.H. Pui, dyhpui@umn.edu, Mechanical Engineering, University of Minnesota, 111 Church St SE, Mechanical Engineering Building, Minneapolis, MN, 55455.

Supplemental Figures and Discussion:

To support the observation that the exposure time affects the number of particles present within a given image, Supplemental Fig. S1 depicts the average particle count per frame as a function of the particle number concentration entering the sensor for four different exposure times. In addition, a linear regression was performed on each of the cases within Supplemental Fig. S1 to aid in the quantitative analysis. These linear regression results are given in Supplemental Table S1.

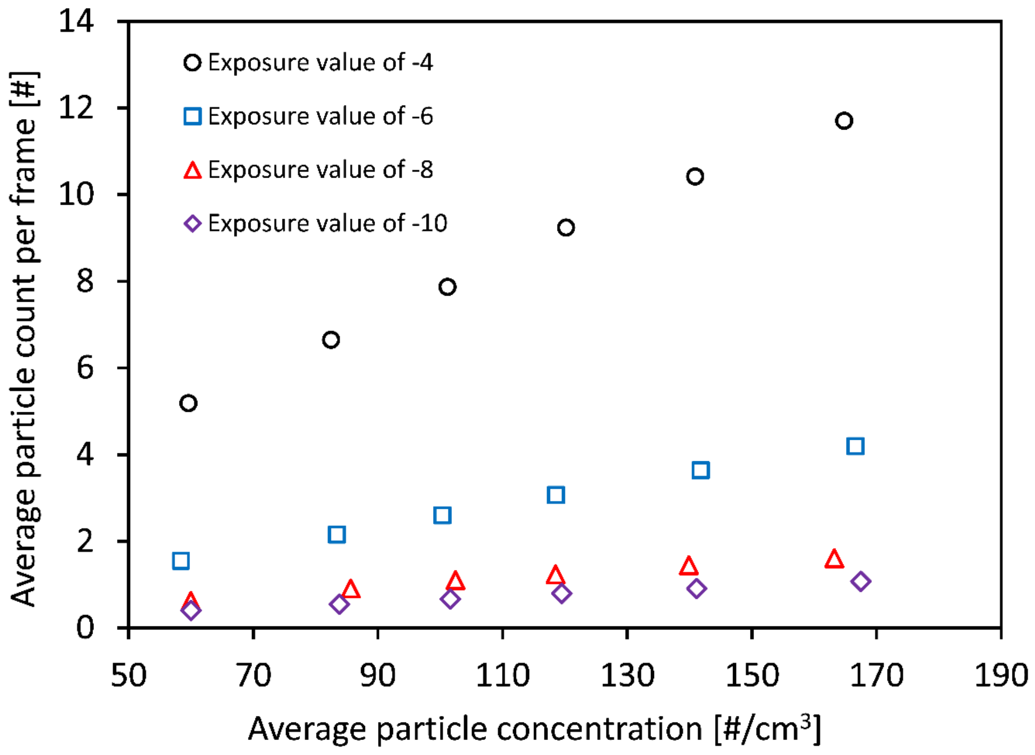

Supplemental Figure S1: Average particle count per frame as a function of the average particle concentration for four different exposure control values. The data points are for 2.87-µm NaCl particles with a 375-nm laser and no filter applied in line with the image sensor. The average flow speed was held constant at 0.64 m/s for all cases. Y-axis error bars are included but are encompassed by datapoints.

Supplemental Table S1: Linear regression values for the four curves given in Supplemental Fig. S1. Confidence intervals are reported at 95% confidence.

Exposure Control Value	Linear Regression Intercept	Linear Regression R^2
-4	1.27 +/- 0.4	0.997
-6	0.11 +/- 0.1	1.000
-8	0.06 +/- 0.1	0.995
-10	0.01 +/- 0.03	0.999

For Supplemental Fig. S1, the average particle counts per frame were calculated based upon 1200 frames, approximately 80 seconds of sampling. The average particle concentrations were calculated by averaging the concentration readings from an Aerodynamic Particle Sizer, Model 3321 APS, during the sampling period. As mentioned in the main document, the higher the exposure value, in this case the closer to zero, the longer the exposure time for each image captured. From the camera manufacturer, the exposure control values of -4, -6, -8, and -10 correspond to exposure times of 62.5 ms, 15.6 ms, 3.9 ms, and 0.98 ms respectively; however, these exposure times were not directly verified.

A longer exposure time will result in more particles being present for a given frame than a shorter exposure time. The longer exposure time allows the particles to travel a greater distance for a constant particle velocity. The greater the distance a given particle can travel, the more likely it is to enter the illuminated area and be captured within the image. This effect leads to an increase in the average number of particles within a given frame, which is observed here. This effect is observed for each of the given exposure control values. The largest average particle counts per frame for a given concentration are for the highest exposure control value whereas the lowest average particle counts per frame for a given concentration are for the lowest exposure control value.

In addition, the shorter the exposure times, the more likely it is that the particles will appear as a small, incomplete signals such as those shown in Fig. 3d. These small incomplete signals could potentially lead to particles within the image not being counted as effectively, leading to a further decrease in the average particles per frame. From visual inspection, this does not appear to be a major issue with the exposure control value of -10 evaluated here, with most particle signals still being accurately counted even if they are incomplete.

For the longer exposure times, while there should be an increased number of particles within the frame, there is also the issue of the increased average pixel value. Usually, this increase in the average pixel value leads to nosier particle counting since non-particle signals will periodically be added to the total count per frame. This effect does appear to be captured by the y-intercept in Supplemental Table S1. The higher the exposure control value, the larger the y-intercept. Since the y-intercept represents the number of particles counted at a particle concentration of 0 $\#/cm^3$, any value above zero signifies some form of particle noise. This value will be larger the worse the noise is. At an exposure control value of -4, this hits 1.27 +/- 0.4 particles per frame, meaning that there is approximately one particle counted per frame that is not physical. For an exposure value of -10, the y-intercept is 0.01 +/- 0.03 signifying few to no false counts from noise.

The y-intercept may not only be increasing due to particle noise though. While the relationship is linear in the concentration region plotted in Supplemental Fig. S1, it has been observed that this linear relationship deviates near the origin. This deviation from the linear relationship at low concentrations would make the y-intercept of the linear portion not fully correspond to the level of noise present in the image at a concentration of 0 $\#/cm^3$. Despite this, it is worth noting this increased intercept as it could potentially signify increased noise for longer exposure times.

A long exposure time can also contribute to overlapping particle signals. Since the particle path through the illuminated area is not strictly controlled, it is possible for two particle signals to overlap one another. For low concentrations and short exposure times, heavy overlapping of particle signals is not observed. For high concentrations and long exposure times, these overlapping particles signals could lead to two particles only being counted as a single particle signal. At the concentrations evaluated, this was not observed to be a major issue; however, it does set an upper limit to the exposure time that can be used and particle

concentrations that can be accurately counted. It is comparable to the coincidence limit for a traditional optical particle counter.

In addition to quantifying the effect of the exposure time on the average particle counts per frame, the effect of the average flow speed on the average particle counts per frame was studied. The results of this are given in Supplemental Fig. S2. The results of the linear regressions for each case are given in Supplemental Table S2.

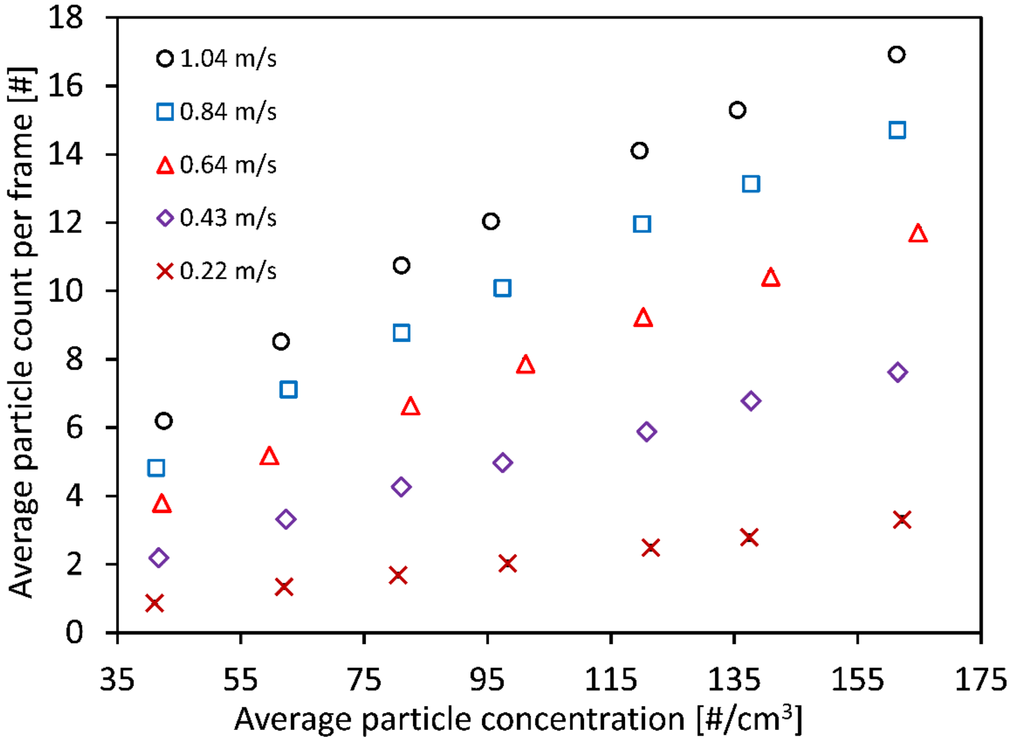

Supplemental Figure S2: Average particle count per frame as a function of the average particle concentration for five different average flow speeds. The data points are for 2.87-µm NaCl particles with a 375-nm laser and no filter applied in line with the image sensor. The exposure control value was held constant at -4 for all cases. Y-axis error bars are included but are encompassed by datapoints.

Supplemental Table S2: Linear regression values for the five curves given in Supplemental Fig. S2. Confidence intervals are reported at 95% confidence.

Average Flow Speed [m/s]	Linear Regression Intercept	Linear Regression R^2
1.04	3.01 +/- 1.4	0.985
0.84	1.9 +/- 0.9	0.993
0.64	1.27 +/- 0.4	0.997
0.43	0.48 +/- 0.4	0.996

0.22	0.09 +/- 0.1	0.999

The average particle count per frame and the average particle concentration were calculated using the same procedure as Supplemental Fig. S1. The average flow speed was calculated based on the inlet flow rate to the sensor and the radius of the inlet. The inlet flow rate was adjusted between 0.5 L/min and 0.1 L/min using a mass flow controller to achieve the velocities depicted in Supplemental Fig. S2.

Supplemental Fig. S2 supports the qualitative observations made with respect to the flow velocity in the main document. The faster the particles and the flow are moving, the more particles on average that can pass through the illuminated area within the given exposure time. This increase in the number of particles passing through the illuminated area will result in more particles being present in each captured image. As observed in Supplemental Fig. S2, the average particle count per frame increases with the increasing average flow speed for all cases. It is important to note that the increased flow speeds also led to an increase in the y-intercept of the average particle count per frame versus concentration curve. As mentioned previously, this increase could reflect an increased level of noise since there should be zero particles counted at a concentration of 0 $\#/cm^3$. The increase in particle counts per frame could be due to this factor; however, a large increase in the noise was not observed visually.

While more particles will pass through the illuminated area, the issue of overlapping particle signals increases as well. Again, the more particles that are captured within a given image, the more likely there will be particles signals that could detected as a single particle instead of multiple. This once again drives the counting efficiency of the particles in the image further from unity. The R^2 value does appear to decrease with increasing average flow speed other than the 0.43-m/s case to the 0.64-m/s case. This decreased linearity could be the result of overlapping particle signals at these higher average flow speeds since this would affect how the particles are counted at low and high concentrations, leading to a deviation from the linear relationship. Additionally, it could be a result of the decreased particle signal from the faster moving particles. This can make counting the particles a more difficult task for the detection algorithm which leads to both undercounting and overcounting. From visual observation, it does appear that the reduced quality of the particle signal is one of the reasons for the decreasing R^2 at the average flow speeds greater than 0.64 m/s. Despite this decrease, the R^2 values show a strong linear relationship in the concentration range evaluated with a value greater than 0.98 for all cases.

While the previous results will apply to bioaerosol detection, they are general trends observed with the image detection method for all counting. To illustrate the quantitative ability to count particles using induced fluorescence, Supplemental Figs. S3 and S4 display the counting of 1% mass riboflavin particles with a 375-nm laser, and a UV-IR cut filter (Gzikai). Additionally, the detection of the same size NaCl particles with and without a UV-IR cut filter (Gzikai) is included to confirm adequate attenuation of the elastic scattering and as a baseline for comparison. Due to the reduced frame rate achievable with the exposure control value used for this data collection, 600 frames were averaged as opposed to 1200 frames during the approximately 80 second sampling period.

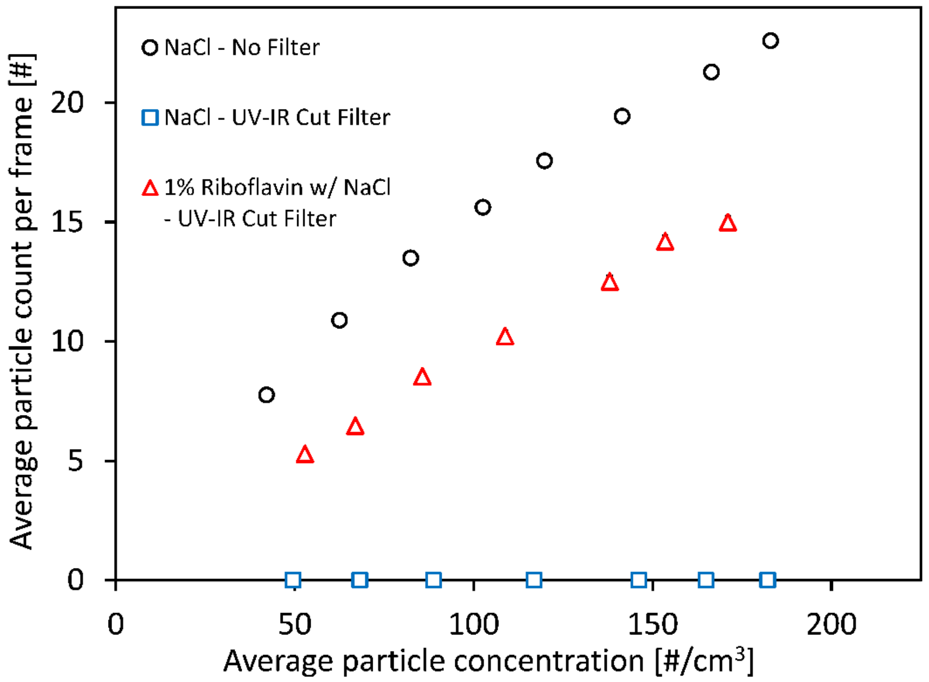

Supplemental Figure S3: Average particle count per frame as a function of the average particle concentration for NaCl particle counting without a filter, NaCl particle counting with a UV-IR cut filter (Gzikai), and 1% mass riboflavin with NaCl particle counting with a UV-IR cut filter (Gzikai). The data points are for 2.87-µm geometric diameter particles with a 375-nm laser, an exposure control value of -3, and an average flow speed of 0.64 m/s. The counting of NaCl particles without a filter is included to illustrate the counting of elastic scattering which is considered the optimal signal strength. The counting of NaCl particles with a filter is included to illustrate attenuation of the elastic scattering.

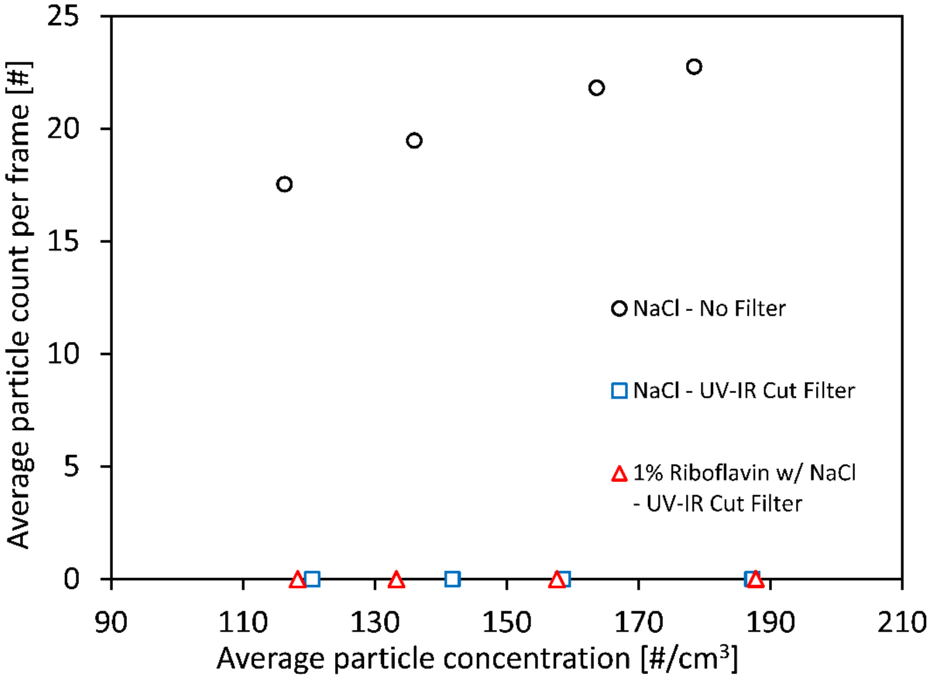

Supplemental Figure S4: Average particle count per frame as a function of the average particle concentration for NaCl particle counting without a filter, NaCl particle counting with a UV-IR cut filter (Gzikai), and 1% mass riboflavin with NaCl particle counting with a UV-IR cut filter (Gzikai). The data points are for 1.33-µm geometric diameter particles with a 375-nm laser, an exposure control value of -3, and an average flow speed of 0.64 m/s. The counting of NaCl particles without a filter is included to illustrate the counting of elastic scattering which is considered the optimal signal strength. The counting of NaCl particles with a filter is included to illustrate adequate attenuation of the elastic scattering.

The counts of the elastic scattering from the NaCl particles were found to be negligible when the UV-IR cut filter was applied, with a maximum of 0.004 counts per frame at a concentration of 182 #/cm^3 for 2.87-µm NaCl particles and 0 counts per frame at a concentration of 187 #/cm^3 for 1.33-µm NaCl particles. From visual inspection, these counts do appear mainly related to noise. In general, this laser wavelength and filter configuration was effective at eliminating the elastic scattering signal, leaving the higher wavelength signals associated with induced fluorescence.

While the induced fluorescence signals were able to be counted at some level, the average counts are lower than the corresponding counts for the elastic scattering detection of NaCl particles with no filter. For Supplemental Fig. S3, the 1% mass riboflavin particles have counts near the purely elastic scattering counting. As mentioned, the elastic scattering provides a brighter particle signal than the induced fluorescence signals. Due to this bright signal, the NaCl elastic scattering detection is used as a reference for the upper limit of counting that can be achieved. This is further supported by the fact that the NaCl elastic scattering detection has roughly size-independent counting between the 2.87-µm and 1.33-µm counting. This signifies that the particle signals from NaCl particles in that size range achieve a sufficient intensity where the particles passing through the illuminated area can be counted consistently. With the 1% mass riboflavin particles reaching counts near the NaCl elastic scattering detection at 2.87 µm, it displays that the detection is nearing this required limit but has not quite achieved it. The elastic scattering counts approximately five particles more for a given concentration than the induced fluorescence case at 2.87 µm.

While the counts are near the elastic scattering detection for 2.87-µm 1% mass riboflavin particles, this is not the case for 1.33-µm particles of the same composition. As mentioned previously, the elastic scattering detection stays relatively constant when decreasing the NaCl particle size from 2.87 µm to 1.33 µm, showing an independence from particle size in this range. For the 1% mass riboflavin particles, moving from a particle size of 2.87 µm to 1.33 µm results in a near complete elimination of particle counts. In general, no 1.33-µm particles of 1% mass riboflavin were able to be counted with this setup of the UV-IR cut filter and a 375-nm laser, except for a handful of particles throughout the sampling period. A real bioaerosol is expected to contain only a fraction of the fluorophore present in the 1% mass riboflavin particles[1]. The signal from a real bioaerosol containing riboflavin will be reduced compared to this detection case.

To display the viability of the color differentiation method, 2.87-um particles of 1% mass riboflavin and NaCl were generated and detected with a 405-nm, 300-mW laser, 2 UV-IR cut filters (Gzikai), and at an average flow speed of 0.1 m/s. Currently, the color difference requires low particle speeds through the viewing area, hence the low average flow speed for this evaluation. The particle counting results are given for both particle compositions in Supplemental Figure S5.

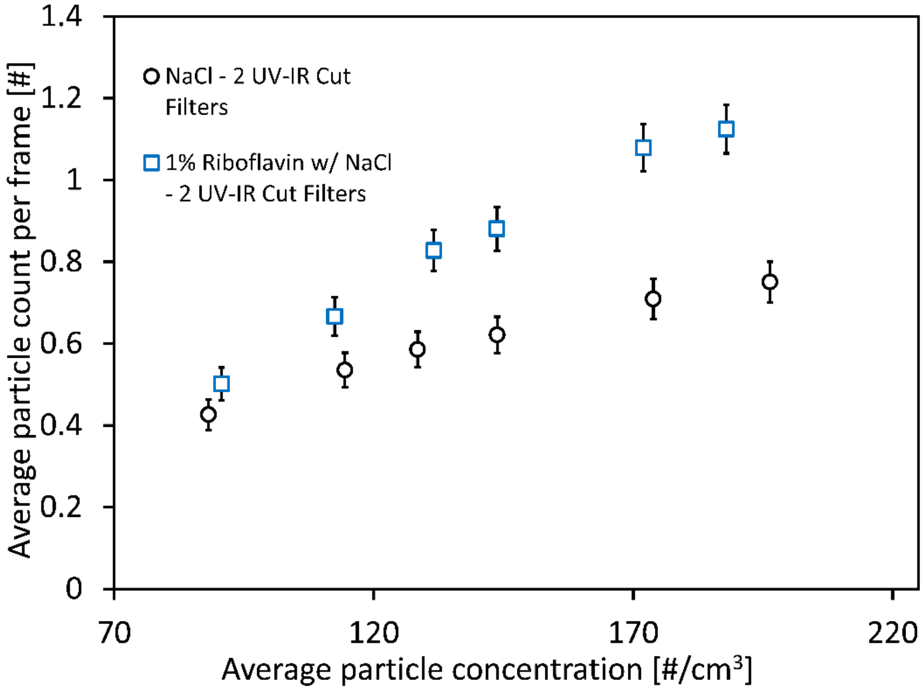

Supplemental Figure S5: Average particle count per frame as a function of the average particle concentration for NaCl particle counting with two UV-IR cut filters (Gzikai), and 1% mass riboflavin with NaCl counting with two UV-IR cut filters (Gzikai). The data points are for 2.87-µm geometric diameter particles with a 405-nm laser, an exposure control value of -4, and an average flow speed of 0.1 m/s. The goal of this method is to allow for the counting of the elastic scattering NaCl particles while differentiating them from particles with an induced fluorescence contribution.

As can be observed, both NaCl particles and 1% mass riboflavin particles can be counted with this optical setup. Note that the counts here are greatly reduced from the previous two figures due to the significant decrease in the average flow speed. While both particle compositions can be counted, the NaCl particles are counted at a reduced rate compared to the 1% mass riboflavin particles. From the slopes of the two curves, it is estimated that the NaCl particles are being counted at approximately 50% the level of the 1% mass riboflavin particles. This reduced counting displays that the elastic scattering contribution is being attenuated too heavily with this optical setup, leading to missed elastic scattering signals. The benefit of this heavy elastic attenuation was differentiable average hues for each particle composition of 224° +/- 1° and 142° +/- 5° for NaCl and 1% mass riboflavin respectively. It will need to be investigated whether the counts for the elastic scattering can be increased while maintaining this discrete color difference. It is important to note that this counting was completed for each particle composition separately, not combined. Further experimental design will be required to test the two compositions in the same detection video.

Finally, to generate the particle diameters evaluated in these supplemental figures, the following parameters in Supplemental Table S3 were used with the Flow-Focusing Monodisperse Aerosol Generator (TSI Model 1520 FMAG)[2]. The dilution flow rate was another critical parameter for the evaluation; however, this quantity was varied to sweep through different particle concentrations for a given size.

Supplemental Table S3: Summary of the particle generation conditions used for each particle size evaluated with the Flow-Focusing Monodisperse Aerosol Generator (TSI Model 1520 FMAG).

Geometric Particle Diameter [μm]	*Solution Concentration*	*Droplet Generation Frequency [kHz]*	*Solution Volumetric Flow Rate [ml/hr]*
2.87	*1.00×10^{-3}*	*90.0*	*4.0*
1.33	*1.00×10^{-4}*	*90.0*	*4.0*

Supplemental References:

1. Hill, S. C. *et al.* Size-dependent fluorescence of bioaerosols: Mathematical model using fluorescing and absorbing molecules in bacteria. *J. Quant. Spectrosc. Radiat. Transf.* **157**, 54–70 (2015).
2. Duan, H. *et al.* Generation of monodisperse aerosols by combining aerodynamic flow-focusing and mechanical perturbation. *Aerosol Sci. Technol.* **50**, 17–25 (2016).
